# Growth, progression and chromosome instability of Neuroblastoma: a new scenario of tumorigenesis?

**DOI:** 10.1186/s12885-016-2986-6

**Published:** 2017-01-05

**Authors:** Gian Paolo Tonini

**Affiliations:** Neuroblastoma Laboratory, Italian Neuroblastoma Foundation, Pediatric Research Institute, Fondazione Città della Speranza, Corso Stati Uniti, 4, 35127 Padua, Italy

**Keywords:** Neuroblastoma, Chromosome instability, Aneuploidy, Genome, Embryo, Neural crest cells

## Abstract

**Background:**

Neuroblastoma is a pediatric cancer with a low survival rate of patients with metastatic stage 4 disease. Tumor aggressiveness and progression have been associated with structural copy number variations (CNVs) that are observed in malignant cells. In contrast, localized Neuroblastomas, which are associated with a low number of structural CNVs but frequent numerical CNVs, are less aggressive, and patients have good outcomes. Finally, whole-genome and whole-exome sequencing of Neuroblastoma tissues have shown few damaging mutations in these tumors.

**Conclusions:**

In the present report it is proposed that chromosome instability (CIN) plays a major role in Neuroblastoma tumorigenesis and that CIN is already present in the early phases of tumor development. High CIN can promote several types of chromosomal damage including chromothripsis, gene deletion, amplification and rearrangements, which deregulate gene expression. Indeed, gene rearrangements have been reported as a new scenario in the development of Neuroblastoma, which supports the hypothesis that CIN is an early step preliminary to the late catastrophic events leading to tumor development.

## Background

### Neuroblastoma

Neuroblastoma, a pediatric cancer, is still a great challenge for oncologists because more than 60% of patients with metastatic disease are not surviving after 5 years from diagnosis. Although a huge amount of clinical and biological data is available, the tumorigenesis of this cancer is largely obscure. The present paper is addressing to stimulate the debate to the tumorigenesis of Neuroblastoma.

Neuroblastoma present as a clinical and biological heterogeneous tumor of embryonic origin that arises from primitive neural crest cells (NCCs) [1]. In about half of the patients this cancer onset as a localized tumor, and their 5-year survival rate ranges between 70% and 98%. The remaining patients present with metastatic stage 4 or stage 4S disease. The latter occurs in approximately 8% of all patients, who are neonates or infants. Usually, these patients have a good outcome. Conversely, stage 4 patients who show an aggressive disease phenotype, have a 5-year survival rate between 30% and 35% [1, 2].

Genome-wide studies have demonstrated that the clinical course of Neuroblastoma is greatly influenced by the presence of copy number variations (CNVs) [3–6]. Numerical CNVs is observed with gain or loss of entire chromosome whereas structural CNVs include gain or loss of partial chromosome. Indeed, many differences in CNVs between localized and metastatic tumors have been reported [4, 7, 8]. Localized tumors display several numerical and few structural CNVs, whereas aggressive metastatic tumors are characterized by a high number of structural CNVs and a low number of numerical CNVs [9–11]. The manner in which CNVs occur during the development of this tumor type is still unknown. The embryonic origin of Neuroblastoma suggests that chromosomal damage is an early event in fetal life. Actually, genomic analyses of tumors in infant patients have revealed several numerical CNVs, which indicate that additional chromosome copies in tumor cells appear very early in the lives of these patients [10, 12, 13]. We can then argue that chromosome mis-segregation may occur early in embryogenesis when NCCs migrate to their final destination.

Since tumors of patients with good survival outcomes primarily have numerical CNVs, while tumors of stage 4 patients with poor outcomes show several structural CNVs, it is acceptable the association between chromosomal structural damage and tumor development and progression. The high number of numerical and structural chromosome disorders observed in Neuroblastoma cells suggests that chromosome instability (CIN) is playing a pivotal role in the tumorigenesis of this tumor type.

Although the CIN is a hallmark of cancer, it has not been extensively explored in Neuroblastoma. Today, a vast amount of genomic data is available, and thus, it would benefit us to turn our attention to the early phases of Neuroblastoma development.

The present report suggests that CIN plays a crucial role in Neuroblastoma tumorigenesis and that CIN characterizes the earliest events in tumor development.

## Main text

### A high number of both numerical and structural CNVs indicates CIN in Neuroblastoma

Clinical observations strongly support the notion that the course of stage 4S Neuroblastoma is initiated with non-aggressive behavior during embryonic life and that this tumor continues to develop during infancy [14] (see also: The European study, LINES 2009 (Low and Intermediate Risk Neuroblastoma European Study), ClinicalTrials.gov Identifier: NCT01728155). This is supported by the fact that tumors of stage 4S patients have several numerical CNVs, whereas stage 4 tumors are characterized by a high number of structural CNVs [10]. Fisher and Tweddle [12] reported a case of neonatal Neuroblastoma in which the tumor showed gains of whole chromosomes as follows: 1, 2, 7, 12, and 17. In our study, which included 30 newborns (aged 0–1 months) with Neuroblastoma, we found that *MYCN* was not amplified in 29/29 (100%) of cases. We also found that chromosome 1p36 was deleted in 1/27 (0.04%) with diploid cells, was not deleted in 12/27 (44%) and was imbalanced in 14/27 (52%) cases. Finally, hyper-diploid DNA content was found in 29/30 (97%) cases (Table 1). Our observation, together with those of other investigators [14–16], confirms that Neuroblastoma can occur during the first year of life and is associated with few genetic aberrations and a favorable clinical stage. In fact, it is interesting to note that 53% (16/30) of patients onset as stage 1; 13% (4/30) as stage 2; 7% (2/30) stage 3; 24% (7/30) stage 4S and only 3% (1/30) stage 4.

**Table 1:** In the Table are listed the 30 patients between 0 and 2 months of life at different neuroblastoma clinical stages. *MYCN* oncogene and chromosome 1p status, and DNA index are also reported

Number^a^	Stage^b^	*MYCN* ^c^	Chromosome 1p^d^	DNA index
1	1	1	imbalance	1.60
2	1	1	imbalance	1.44
3	1	1	imbalance	1.37
4	1	1	imbalance	1.64
5	1	1	normal	1.49
6	1	1	normal	2.09
7	1	1	imbalance	1.31
8	1	1	imbalance	1.35
9	1	1	normal	1.60
10	1	1	normal	1.77
11	1	1	normal	1.85
12	1	1	normal	1.19
13	1	1	normal	1.21
14	1	1	normal	1.08
15	1	1	n.d.	1.36
16	1	n.d.	n.d.	1.21
17	2	1	imbalance	1.23
18	2	1	imbalance	1.50
19	2	1	imbalance	1.63
20	2	1	imbalance	1.52
21	3	1	normal	1.29
22	3	1	normal	1.47
23	4	1	n.d.	1.17
24	4S	1	imbalance	2.33
25	4S	1	loss	1.00
26	4S	1	normal	1.30
27	4S	1	imbalance	1.55
28	4S	1	imbalance	1.40
29	4S	1	imbalance	1.65
30	4S	1	normal	1.20

^a^Serial number; ^b^Clinical stage; ^c^one copy of *MYCN* gene; ^d^normal: normal chromosome 1p; imbalance: extracopy of chromosome 1p; loss: loss of chromosome 1p; *n.d* not done. (Data from Italian Neuroblastoma Register [2])

**Fig. 1:**
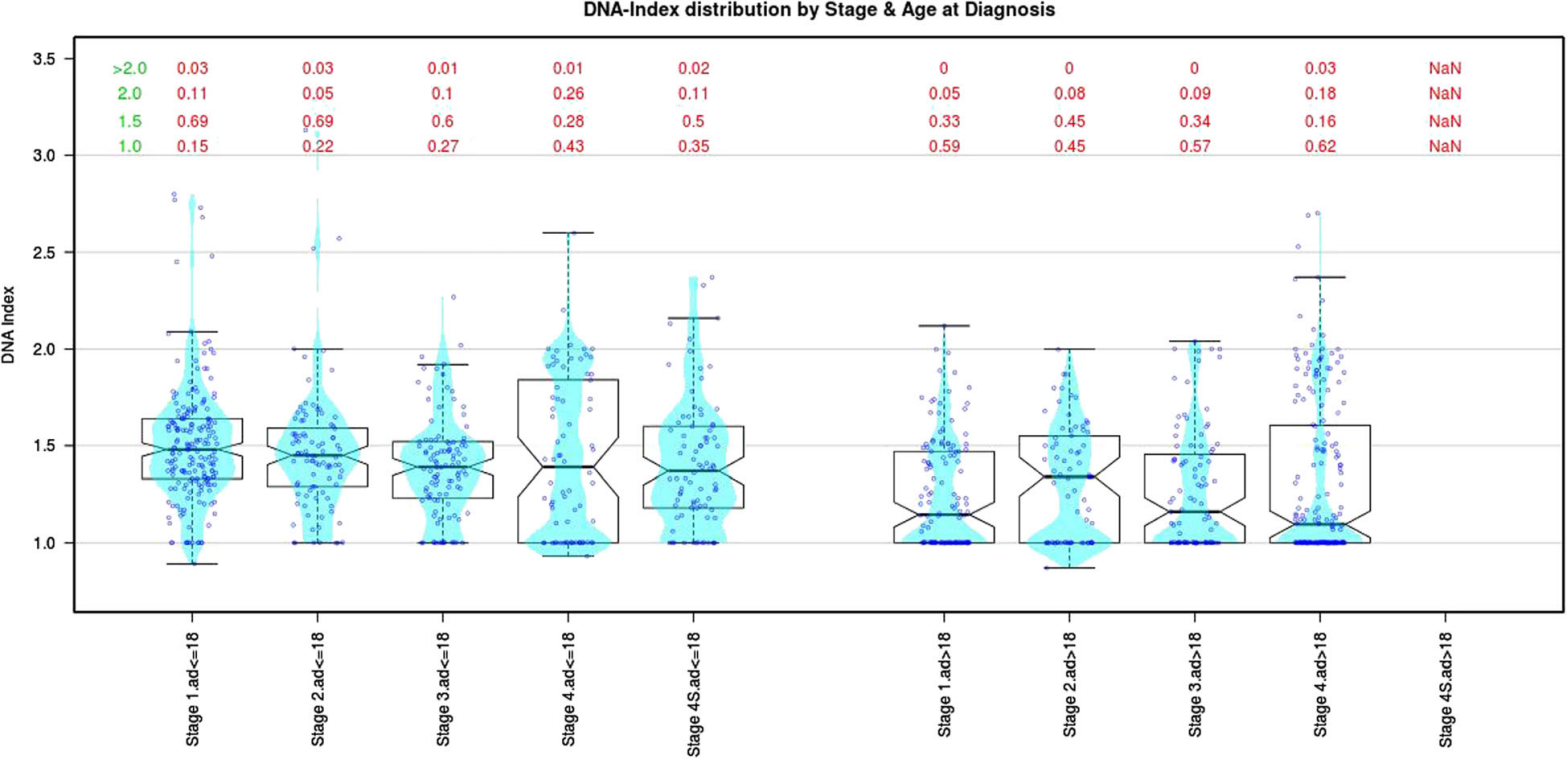
Distribution of the DNA index value of 2994 neuroblastoma tumors belonging to Italian Neuroblastoma Register [2]. The DNA index percentage value for each clinical stage is reported in the upper panel (*red* color). On the left, green value are referred to the 1.0 for diploid cells, 1.5 and 2.00 for hyperdiploid cells. A DNA index of 1.50 is more frequent in tumors of patients who are younger than 18 months for all stages but not for stage 4 patients who have a peak of 43% DNA of diploid cells. Tumors of patients over 18 months of age primarily show a DNA diploid tumor cells with a peak (62%) of tumor cells in stage 4. Stage 4S is not represented because occurs in patients under 12 months of age, by definition

It is widely accepted that near-triploid cells are characteristic of low-risk Neuroblastoma, which indicates that supernumerary whole chromosomes are a feature of non-aggressive tumors [15, 17]. Recently, we performed a survey of 2994 cases, which included all stages of Neuroblastoma, and we confirmed that aneuploidy is more frequent in tumors of patients younger than 18 months of age with stage 1, 2, 3, or 4 disease compared with older patients (Fig. 1). Conversely, the tumors of children older than 18 months are primarily near-diploid or near-tetraploid, and this feature is independent of the patient’s clinical stage; although, this characteristic is most evident in stage 4 Neuroblastoma. In particular, tumor with DNA index 1.5 has been found in younger patients belonging to: stage 1, 69%; 2, 69%; 3, 60; 4, 28%; 4S, 50%. On the contrary, the percentage of tumor with DNA index 1.5 is lower in the patients older than 18 months according the following stages: 1, 33%; 2, 45%; 3, 34%; 4, 16%. It is to note that tumor cells with diploid DNA content is growing from stage 1 to 4 as well as in patients under and over 18 months of age.

It is noteworthy that near-diploid tumors of high-risk patients with stage 4 disease contain several structural CNVs. As observed by Brodeur et al. [18] “segmental CNVs do not substantially alter the total cellular DNA content”, which indicates that tumors of high-risk patients contain several chromosomal aberrations, although with near-diploid DNA content. Aneuploidy is generally defined as the presence of an abnormal chromosome number that is greater or smaller than the diploid component. Duesberg and Li [19] defined aneuploidy “as an abnormal balance of either intact chromosomes or segments of chromosomes or both”. Besides, Geigl et al. [20] defined aneuploidy as an “unbalanced number of chromosomes or large portions of chromosomes”. All above suggests that aneuploid Neuroblastoma cells have unequal chromosome content not only because of entire chromosome gains but also because of the gain and/or loss of partial chromosome regions.

On the whole, genome-wide studies have demonstrated that critical chromosomal damage occurs more frequently in older patients and that several CNVs accumulate in an age-dependent manner, as supported by Schleiermacher et al. [9] and Coco et al. [10].

Finally, as additional help to the facts discussed above, it is interesting to note that cancer mouse models provide the opportunity to demonstrate that CIN can initiate tumor transformation [21].

### CIN is present in the early phases of Neuroblastoma development

Geigl et al. [20] described CIN as “…the rate (cell-to-cell variability) of gain or loss of whole chromosomes or fractions of chromosomes. This definition encompasses the rate of both whole-chromosome and segmental chromosomal aneuploidies”. Taking into account the Geigl’s sentence, I suggest that CIN is a feature of Neuroblastoma as indicated by the present of both numerical and structural CNVs in Neuroblastoma cells. Abnormal DNA content in Neuroblastoma cells may be triggered by aberrant mitosis, chromosome mis-segregation, and kinetochore defects as well as is occurring in many cancers [22–24]. Indeed, both chromosome mis-segregation and kinetochore abnormalities produce an unequal number of chromosomes in daughter cells. Therefore, it may be reasonable to suppose that this represents one of the first genomic catastrophic events in premalignant Neuroblastoma cells. A popular hypothesis to explain the development of aneuploidy postulates that polyploidization may be caused by multiple rounds of S-phase in the absence of mitotic endoreduplication [25]. The subsequent and progressive loss of chromosomes from the original polyploid progenitor cell and subsequent rearrangements within the extra-copy genetic reservoir would generate aneuploid cells. Another mechanism that may be responsible for aneuploidy in Neuroblastoma cells might be associated with the abnormal activity of the spindle apparatus [26]. It has been reported that chromosome mis-segregation leads to DNA damage, particularly chromosome translocation [27]. Finally, we may also add that chromothripsis, which has been recently observed in Neuroblastoma [28], indicates a high CIN in this tumor.

It is noteworthy that structural chromosomal damage as well as large and small DNA sequence rearrangements are associated with deregulated transcription. Therefore, Neuroblastoma cells have both chromosomal and transcriptional instability [11, 29, 30].

### Neuroblastoma and CIN during embryonic development

**Fig. 2:**
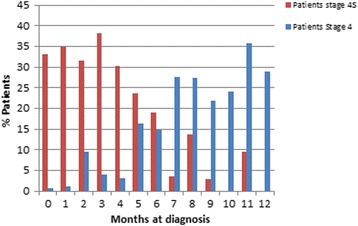
Distribution of stage 4 and stage 4S Italian patients from the National Neuroblastoma Register according to the age of the patients at diagnosis. Most of the stage 4S cases are observed between 0 and 6 months. On the contrary, the frequency of stage 4 cases increases after 6 months of observation

Approximately 5% of Neuroblastoma are detected during the neonatal period, which indicates that this type of neoplasia likely grows during the embryonic period [31]. In necropsies of infants, Ikeda et al. [32] observed nodules of Neuroblastoma cells, which they defined as “*in situ* neuroblastoma”. These nodules provide evidence that Neuroblastoma may be present in the embryo. Moreover, most patients experience the onset of stage 4S disease within 6 months of birth (Fig. 2), which suggests that the initiation of this tumor occurs during fetal development. This concept has been further supported by Gigliotti et al. [14] who reported that 6 cases out of 45 stage 4S Neuroblastoma were detected *in utero*.

Within the last decade, the study of chromosome instability during early embryogenesis has become possible with the introduction of preimplantation genetic diagnosis (PGD) along with the in vitro fertilization (IVF) technique [33, 34]. Surprisingly, mosaicism of whole-chromosome aneuploidies and uniparental disomies, as well as segmental deletions, duplications and amplifications, are not restricted to arrested or poorly developed cleavage-stage embryos, but are also common in high-quality IVF embryos.

The presence of CIN in fetal tissues supports the notion that chromosome instability may persist for a longer period during fetal life. In particular, we could argue that neural crest cells with high CIN could generate neuroblastic pre-malignant cells with abnormal chromosome content. This phenomenon might be a leading cause of numerical and structural CNVs in Neuroblastoma cells.

### CIN may explain the tumorigenesis of Neuroblastoma

The route of Neuroblastoma development appears complex; several genomic damages justify that NCCs can undergo to malignant transformation during the embryonic life. However, to build a confident model for Neuroblastoma tumorigenesis is very difficult. The Neuroblastoma shows high clinical and biological heterogeneity indicating that tumorigenesis is complex and probably involve several genetics aspects. In 1976, Knudson and Meadows [35, 36] proposed the two-step mutation model to explain the evolution of stage 4S suggesting the presence of a single recessive gene mutation. A model that should also explained the tumor spontaneous regression. However, the two-step model did not fit with the other clinical stages of Neuroblastoma and was never proved for stage 4S. Afterwards, numerous studies have revealed several genetics abnormalities in Neuroblastoma such as *MYCN* gene amplification, numerical chromosome abnormalities and non-random chromosome gains and losses. In 1993, I suggested that more genes and chromosome abnormalities participated in a multistep manner to the Neuroblastoma tumorigenesis [37, 38]. Unfortunately, the model did not explained how the genetics abnormalities are occurring sequentially.

More recently, we used array comparative genomic hybridization datasets and a dictionary-learning algorithm to develop a progressive genomic instability model of metastatic Neuroblastoma tumorigenesis [39]. This model supports the hypothesis that the initial step of oncogenesis is the generation of whole chromosome gains followed by loss of chromosome segments. The progressive genomic instability model together the observation of Coco et al. [10] supports the role of CIN in Neuroblastoma and justifies the observation that tumors in newborns have numerical chromosome gains while those of older patients have more structural chromosome aberrations indicating the condition of high genomic instability.

## Conclusions

### Neuroblastoma: a CIN disease

In conclusion, currently, sufficient biological, molecular and clinical observations have been made, which allowed us to hypothesize that the embryonic development of Neuroblastoma is a consequence of high chromosome instability of neural crest cells. In my opinion, metastatic stage 4S and stage 4 cells originate from a common ancestral neural crest cell. The accumulation of structural CNVs in stage 4 tumors, but not in 4S tumors, depends on the time necessary for these critical impairments to occur in the cells [10]. Thus, the tumor cells of patients who are older than one year of age with stage 4 disease have had sufficient time to accumulate several structural CNVs.

The role of point mutations in this process is questionable. For instance, deleterious mutations in genes whose products comprise the spindle apparatus could disable these genes and prevent normal regulation of chromosome segregation in neural crest cells. Actually, recurrent mutations rarely occur in the Neuroblastoma genome. Pugh et al. [40] selected only a few genes with missense mutations. Among the most frequently mutated genes, *ALK* ranks first with 22 missense mutations. Furthermore, Pugh’s results confirmed the low number of missense mutations (12 per tumor) observed in Neuroblastoma [28]. Moreover, Molenaar et al. [28] reported that the frequency of mutations in stage 4S tumors is less than 5%. Similarly, we showed that *RAS*, a gene that is often mutated in adult cancers and in leukemia, is rarely mutated in Neuroblastoma [41]. Additionally, the *ATRX* [40] gene, a putative driver gene in the oncogenesis of Neuroblastoma, shows a gene deletion, but few mutations.

More recently, Peifer et al. [42] confirmed a low mutation rate in Neuroblastoma as reported by Molenaar et al. [28]. It is interesting to note that the same author showed that telomerase genes are over expressed in high-risk Neuroblastoma as a consequence of genomic rearrangements. Peifer and colleagues conclude that genome remodeling is the cause of telomerase gene activation. This finding supports the hypothesis that genome instability is an early event in Neuroblastoma tumorigenesis.

In conclusion, the large amount of genomic data on Neuroblastoma and several clinical observations in regards to the natural history of the disease allow us to propose that Neuroblastoma arises due to the chromosome instability present in neural crest cells. If we view Neuroblastoma as a CIN disease, we may use drugs that target CIN to improve the treatment of this tumor. Indeed, some compounds such as Aurora A inhibitors are already being assessed in preclinical and clinical studies of Neuroblastoma therapy [43].

## Abbreviations

CIN: Chromosome instability
CNVs: Copy number variations
IVF: In vitro fertilization
NCCs: Neural crest cells
PGD: Preimplantation genetic diagnosis

## References

[1] Luksch R, Castellani MR, Collini P, De Bernardi B, Conte M, Gambini C, Gandola L, Garaventa A, Biasoni D, Podda M, Sementa AR, Gatta G, Tonini GP. Neuroblastoma (Peripheral neuroblastic tumours). Crit Rev Oncol Hematol. 2016;107:163-81. 10.1016/j.critrevonc.2016.10.001 27823645

[2] Haupt R, Garaventa A, Gambini C, Parodi S, Cangemi G, Casale F, Viscardi E, Bianchi M, Prete A, Jenkner A (2010). Improved survival of children with neuroblastoma between 1979 and 2005: a report of the Italian Neuroblastoma Registry. J Clin Oncol.

[3] Janoueix-Lerosey I, Schleiermacher G, Michels E, Mosseri V, Ribeiro A, Lequin D, Vermeulen J, Couturier J, Peuchmaur M, Valent A (2009). Overall genomic pattern is a predictor of outcome in neuroblastoma. J Clin Oncol.

[4] Schleiermacher G, Michon J, Ribeiro A, Pierron G, Mosseri V, Rubie H, Munzer C, Bénard J, Auger N, Combaret V (2011). Segmental chromosomal alterations lead to a higher risk of relapse in infants with MYCN-non-amplified localised unresectable/disseminated neuroblastoma (a SIOPEN collaborative study). Br J Cancer.

[5] Defferrari R, Mazzocco K, Ambros IM, Ambros PF, Bedwell C, Beiske K, Benard J, Berbegall AP, Bown N, Combaret V (2015). Influence of segmental chromosome abnormalities on survival in children over the age of 12 months with unresectable localised peripheral neuroblastic tumours without MYCN amplification. Br J Cancer.

[6] Tonini GP, Romani M (2003). Genetic and epigenetic alterations in neuroblastoma. Cancer Lett.

[7] Scaruffi P, Coco S, Cifuentes F, Albino D, Nair M, Defferrari R, Mazzocco K, Tonini GP (2007). Identification and characterization of DNA imbalances in neuroblastoma by high-resolution oligonucleotide array comparative genomic hybridization. Cancer Genet Cytogenet.

[8] Ohira M, Nakagawara A (2010). Global genomic and RNA profiles for novel risk stratification of neuroblastoma. Cancer Sci.

[9] Schleiermacher G, Janoueix-Lerosey I, Ribeiro A, Klijanienko J, Couturier J, Pierron G, Mosseri V, Valent A, Auger N, Plantaz D (2010). Accumulation of segmental alterations determines progression in neuroblastoma. J Clin Oncol.

[10] Coco S, Theissen J, Scaruffi P, Stigliani S, Moretti S, Oberthuer A, Valdora F, Fischer M, Gallo F, Hero B (2012). Age-dependent accumulation of genomic aberrations and deregulation of cell cycle and telomerase genes in metastatic neuroblastoma. Int J Cancer.

[11] Stigliani S, Coco S, Moretti S, Oberthuer A, Fischer M, Theissen J, Gallo F, Garavent A, Berthold F, Bonassi S (2012). High genomic instability predicts survival in metastatic high-risk neuroblastoma. Neoplasia.

[12] Fisher JP, Tweddle DA (2012). Neonatal neuroblastoma. Semin Fetal Neonatal Med.

[13] Brodeur GM (2003). Neuroblastoma: biological insights into a clinical enigma. Nat Rev Cancer.

[14] Gigliotti AR, Di Cataldo A, Sorrentino S, Parodi S, Rizzo A, Buffa P, Granata C, Sementa AR, Fagnani AM, Provenzi M (2009). Neuroblastoma in the newborn. A study of the Italian Neuroblastoma Registry. Eur J Cancer.

[15] Taggart DR, London WB, Schmidt ML, DuBois SG, Monclair TF, Nakagawara A, De Bernardi B, Ambros PF, Pearson AD, Cohn SL (2011). Prognostic value of the stage 4S metastatic pattern and tumor biology in patients with metastatic neuroblastoma diagnosed between birth and 18 months of age. J Clin Oncol.

[16] Rubie H, De Bernardi B, Gerrard M, Canete A, Ladenstein R, Couturier J, Ambros P, Munzer C, Pearson AD, Garaventa A (2011). Excellent outcome with reduced treatment in infants with nonmetastatic and unresectable neuroblastoma without MYCN amplification: results of the prospective INES 99.1. J Clin Oncol.

[17] George RE, London WB, Cohn SL, Maris JM, Kretschmar C, Diller L, Brodeur GM, Castleberry RP, Look AT (2005). Hyperdiploidy plus nonamplified MYCN confers a favorable prognosis in children 12 to 18 months old with disseminated neuroblastoma: a Pediatric Oncology Group study. J Clin Oncol.

[18] Brodeur GM, Hogarty MD, Mosse YP, Maris JM, Pizzo PA, Poplack DG (2011). Neuroblastoma. Principles and practice of pediatric oncology.

[19] Duesberg P, Li R (2003). Multistep carcinogenesis: a chain reaction of aneuploidizations. Cell Cycle.

[20] Geigl JB, Obenauf AC, Schwarzbraun T, Speicher MR (2008). Defining ‘chromosomal instability’. Trends Genet.

[21] Schvartzman JM, Sotillo R, Benezra R (2010). Mitotic chromosomal instability and cancer: mouse modelling of the human disease. Nat Rev Cancer.

[22] Ganem NJ, Storchova Z, Pellman D (2007). Tetraploidy, aneuploidy and cancer. Curr Opin Genet Dev.

[23] Kops GJ, Weaver BA, Cleveland DW (2005). On the road to cancer: aneuploidy and the mitotic checkpoint. Nat Rev Cancer.

[24] Russo A, Pacchierotti F, Cimini D, Ganem NJ, Genesca A, Natarajan AT, Pavanello S, Valle G, Degrassi F (2015). Genomic instability: Crossing pathways at the origin of structural and numerical chromosome changes. Environ Mol Mutagen.

[25] Pihan GA, Doxsey SJ (1999). The mitotic machinery as a source of genetic instability in cancer. Semin Cancer Biol.

[26] Ooi WF, Re A, Sidarovich V, Canella V, Arseni N, Adami V, Guarguaglini G, Giubettini M, Scaruffi P, Stigliani S (2012). Segmental chromosome aberrations converge on overexpression of mitotic spindle regulatory genes in high-risk neuroblastoma. Genes Chromosomes Cancer.

[27] Janssen A, van der Burg M, Szuhai K, Kops GJ, Medema RH (2011). Chromosome segregation errors as a cause of DNA damage and structural chromosome aberrations. Science.

[28] Molenaar JJ, Koster J, Zwijnenburg DA, van Sluis P, Valentijn LJ, van der Ploeg I, Hamdi M, van Nes J, Westerman BA, van Arkel J (2012). Sequencing of neuroblastoma identifies chromothripsis and defects in neuritogenesis genes. Nature.

[29] Xiong Y, Wu S, Du Q, Wang A, Wang Z (2015). Integrated analysis of gene expression and genomic aberration data in osteosarcoma (OS). Cancer Gene Ther.

[30] Yang Z, Zhuan B, Yan Y, Jiang S, Wang T (2015). Integrated analyses of copy number variations and gene differential expression in lung squamous-cell carcinoma. Biol Res.

[31] Dhir S, Wheeler K (2010). Neonatal neuroblastoma. Early Hum Dev.

[32] Ikeda Y, Lister J, Bouton JM, Buyukpamukcu M (1981). Congenital neuroblastoma, neuroblastoma in situ, and the normal fetal development of the adrenal. J Pediatr Surg.

[33] Vanneste E, Voet T, Le Caignec C, Ampe M, Konings P, Melotte C, Debrock S, Amyere M, Vikkula M, Schuit F (2009). Chromosome instability is common in human cleavage-stage embryos. Nat Med.

[34] Vanneste E, Melotte C, Voet T, Robberecht C, Debrock S, Pexsters A, Staessen C, Tomassetti C, Legius E, D’Hooghe T (2011). PGD for a complex chromosomal rearrangement by array comparative genomic hybridization. Hum Reprod.

[35] Knudson AG, Meadows AT (1976). Developmental genetics of neuroblastoma. J Natl Cancer Inst.

[36] Knudson AG, Meadows AT (1980). Sounding board. Regression of neuroblastoma IV-S: a genetic hypothesis. N Engl J Med.

[37] Tonini GP (1993). Neuroblastoma: a multiple biological disease. Eur J Cancer.

[38] Tonini GP (1993). Neuroblastoma: the result of multistep transformation?. Stem Cells.

[39] Masecchia S, Coco S, Barla A, Verri A, Tonini GP (2015). Genome instability model of metastatic neuroblastoma tumorigenesis by a dictionary learning algorithm. BMC Med Genomics.

[40] Pugh TJ, Morozova O, Attiyeh EF, Asgharzadeh S, Wei JS, Auclair D, Carter SL, Cibulskis K, Hanna M, Kiezun A (2013). The genetic landscape of high-risk neuroblastoma. Nat Genet.

[41] Iolascon A, Badiali M, Pession A, Basso G, Losi L, Delgiudice E, Perrotta S, Cutillo S, Tonini G (1993). Rare frequencey of point mutations for codon 12, 13 and 61 of ras gene in italian neuroblastoma. Int J Oncol.

[42] Peifer M, Hertwig F, Roels F, Dreidax D, Gartlgruber M, Menon R, Kramer A, Roncaioli JL, Sand F, Heuckmann JM (2015). Telomerase activation by genomic rearrangements in high-risk neuroblastoma. Nature.

[43] Niu H, Manfredi M, Ecsedy JA (2015). Scientific Rationale Supporting the Clinical Development Strategy for the Investigational Aurora A Kinase Inhibitor Alisertib in Cancer. Front Oncol.

